# The Examination of Fatigued Children

**Published:** 1915-11-06

**Authors:** 


### THE SCHOOL DOCTOR.

### THE EXAMINATION OF FATIGUED CHILDREN.

A great deal has been written in late years about fatigue in school children, and undoubtedly the subject is of high interest both from a practical and from a theoretical point of view. Briefly put, it may be stated that fatigue in children, as in adults, shows itself in two ways: as mental fatigue and as physical fatigue. The distinction between these two kinds is more arbitrary than real; with all mental fatigue states there exists a certain amount of fairly evident physical or tissue fatigue; with all physical fatigue states there appears a corresponding, though not quantitatively similar, degree of mental fatigue. Yet in practice the distinction between these two kinds of fatigue or tiredness should be remembered, even though, strictly speaking, it cannot be scientifically upheld.

The evidence of the existence of fatigue is found in the existence of partial or general tiredness; in other words, in a greater or less inability to respond to normal stimulus. It is stated as a physiological axiom that nerve tissue cannot be fatigued, but this statement does not hold good for practical purposes. When nerve tissue is excited or put into action certain degenerative changes are observed, and it is now fairly clear that such changes throw into the blood-stream definite toxins or chemical decomposition products that act as tissue tirers. Much experimental work on the subject of so-called kenotoxins has shown that these poisonous products can be isolated, and when injected into the unfatigued organism rapidly succeed in producing a high degree of fatigue without the help of active work on the part of the tissues themselves.

### How to Diagnose Fatigue.

For the school doctor's purpose, however, the main thing is how to diagnose fatigue and how to elucidate its probable causation in certain children. The first problem is generally easy. We are all familiar with the signs of tiredness in children; the drooping head, lack-lustre eye, and general appearance of drowsiness tell their tale quite easily. But they are evidence of a rather high degree of tissue fatigue, and the inspector should accustom himself to diagnose lesser states of fatigue so as to be able to adopt precautionary measures in the case of children who are more likely to be fatigued through normal school work than their fellow class-mates. Among the finer tests of tissue tiredness are the skin reflexes and the response made by the tissues to slight stimulus. The width of the pupil, for instance, is a good sign. In a fatigued child it is dilated, and it responds slowly to external stimuli, not so slow, it is true, as in pathological states, but not with the same rapidity of response that a normal child shows. Another good test is the "tickling" test; most children—though this is a moot point—respond quickly to slight tickling in the flanks; the fatigued child scarcely responds at all. Other valuable signs are a fall in the blood pressure, and slight and transient signs of inco-ordination and tremor. Indeed, there are about 600 scientific tests to prove the existence of slight signs of fatigue in children, but the simple ones given above are for all practical purposes sufficient to enable the inspector to make a diagnosis.

### Causes of Fatigue.

Having discovered the fact that a certain child is fatigued, it is then the inspector's duty to try to find out what is the cause of such fatigue. If there is evidence of a body lesion, however slight that may be, this may be accepted as almost sufficient explanation of the fatigue. It is striking to find what apparently insignificant degrees of defectiveness are sufficient in themselves to cause a fairly large amount of fatigue in young children. The sepsis derived from one carious tooth, from constipation, from chronic appendicitis, from chronic middle-ear disease, and from septic tonsils, rapidly produces fatigue in children who suffer from such defects; such children are more easily tired by ordinary class work that does not throw a great strain on the normal child; they assimilate knowledge less easily; the teacher finds them dull and inattentive, whereas they are merely ill. When these defects have been treated it is generally found that the children rapidly make up for ground lost in the class, and become alert and intelligent pupils, so much so that the teachers often express their astonishment at the change. The good effects following the simple operative treatment of adenoids and tonsils in some school children are undoubtedly due to this elimination of fatigue-producing factors.

### Faulty Methods of Teaching.

By far the most common cause of fatigue in school children is defective physique and tissue sepsis. When a defect has been discovered it must be remedied before the doctor can state that the fatigue from which the child suffers was not caused thereby. But sometimes, and by no means rarely, we find pronounced signs of fatigue in children who are physically normal, and in whom no trace of tissue sepsis can be discovered. We have then to look for other causative factors. One of these is undoubtedly faulty methods of teaching. The average child has a blessed asset that guards him against fatigue when he is cursed with a prosy teacher; he rapidly gets bored and his attention wanders; the conscientious child who attempts to follow the teacher and does not allow his mind to wander succumbs to the strain and becomes fatigued. Keats described the process very well in his recollections of the lectures he used to attend; he allowed his mind to wander into space and speculated upon the fairies travelling on the motes of dust that shimmered in a sunbeam in the class-room. Against faulty methods of teaching the school doctor can do little; but he can impress their importance as fatigue-causing factors upon the teachers in a friendly fashion, in the hope that his words may cause some mental reflection and future reformation.

Equally potent a cause of fatigue in school children who are physically normal is want of sleep and overstrain. This may be due to many causes. In London, and generally in city districts, the most common cause of want of sleep, apart from late hours and picture shows, is vermin. Fleas and bed lice worry the child so that he cannot drop off to sleep easily; evidence of their activity can usually be found on examining the children's skins and clothing. A very frequent cause of fatigue is undoubtedly scouting; few scout-masters understand the hygiene of children, and it is an unfortunate fact that most scout practices are held in the evenings, so that the boys go to bed late. Choir practice is another potent cause. Home-work, although frequently indicted, is a far less frequent cause; but teachers should be urged to discriminate in apportioning home tasks. Emotional causes undoubtedly create fatigue; so do school games, but their influence has been much exaggerated. In general it may be stated that a normal child will not be fatigued by ordinary class work, nor by the usual play enjoyed by children of school-going age.

The treatment of fatigue must aim in the first place at removing the cause. The teacher or parent must be told what is the matter, and must be urged to adopt precautionary measures suitable to the case. Where vermin are the cause the school nurse must be asked to visit the home and make a report, and action must be taken if there is what lawyers call "contributory negligence" on the part of the parents. Where a definite physical defect exists this must be treated as a preliminary to any other kind of treatment that may be advised. Open air, food, and variety of task work or recreation will be found better than medicine or tonics in the majority of other cases where the fatigue depends not so much upon tissue exhaustion as upon improper use of body or brain.

